# System-Level Design and Integration of Dexterous Robotic Hands for Complex Manipulation

**DOI:** 10.34133/research.1388

**Published:** 2026-08-12

**Authors:** Qianqian Wang, Qijun Yang, Xuanyu An, Yang Liu, Wei Zhang, Elahe Abdi, Liying Tian, Zhiqiang Tang

**Affiliations:** ^1^ Jiangsu Key Laboratory for Design and Manufacturing of Precision Medicine Equipment, School of Mechanical Engineering, Southeast University, Nanjing 211189, China.; ^2^ GLI Technology Limited, Shenzhen, China.; ^3^ School of Materials Science and Engineering, Southeast University, Nanjing, China.; ^4^ Department of Mechanical and Aerospace Engineering, Monash University, Melbourne, Australia.; ^5^ School of Materials Science and Engineering Nanyang Technological University, Nanyang Avenue, Singapore, Singapore.

## Abstract

Driven by the demands of intelligent manufacturing, medical rehabilitation, and human–robot collaboration, anthropomorphic dexterous hands have become a key direction for complex manipulation. This paper reviews their evolution from structural biomimetics to intelligent control, emphasizing structural optimization, task generalization, and perception–control coupling. First, it summarizes structural design principles, including structural parameterization, underactuated and compliant mechanisms, functional materials, and multimaterial fabrication. Second, it reviews perception technologies, including angle and strain sensing, tactile and pressure detection, and compact vision–tactile fusion modules. Third, it compares classical model-based control, data-driven learning control, and hybrid model–data control, with attention to sample efficiency, robustness, and transferability. Finally, it discusses deployment-oriented strategies, including modular design, standardized interfaces, integrated packaging, and unified communication frameworks for hardware–software coordination. In summary, this paper establishes a system-level technical framework for dexterous hand development and deployment, organized around 4 tightly coupled dimensions: structural embodiment, multimodal perception, control, and deployment-oriented integration. By clarifying the interactions and constraints across these dimensions, the paper provides a system-level basis for the design, evaluation, and deployment of dexterous hands in complex manipulation and human–robot collaboration.

## Introduction

The human hand is one of the most complex biological structures. Its remarkable dexterity arises from a multilayered anatomical design, compliant materials, and a highly refined neural control system [1–6]. Collectively, the bones, joints, tendons, skin, and nerves constitute a highly articulated system characterized by abundant degrees of freedom (DOF), intrinsic adaptability, and rich sensory feedback. This enables precise control of various objects in both static grasping and dynamic manipulation [7]. The opposable structure formed by the thumb and the other 4 fingers grants humans a unique advantage in tool use and environmental adaptation. Such synergy between structural design and neural control has become a key biological foundation and source of inspiration for anthropomorphic robotic hands [8].

In recent years, advances in neurophysiology have expanded the modeling of the human hand beyond its anatomical structure, emphasizing integrated neuromuscular-skeletal coordination. This perspective provides theoretical support for the design of robotic structures, sensor integration, and control strategies. As a result, the design paradigm has gradually shifted from mimicking physical form to reconstructing functional capability. This shift now defines a central trajectory in the development of anthropomorphic dexterous hands.

Building on this shift in design philosophy, the role of anthropomorphic robotic hands has gained increasing prominence in the broader context of robotics [9,10]. With the rapid development of artificial intelligence, intelligent manufacturing, and human–robot collaboration, the pursuit of human-level dexterous manipulation has become a key objective in the field. This capability is essential for enabling robots to perform complex, human-like tasks in dynamic environments. Among the various approaches to achieving this goal, anthropomorphic robotic hands have emerged as one of the most promising solutions. These systems embody both structural and functional resemblance to the human hand. More importantly, they demonstrate autonomy and adaptability when dealing with diverse and challenging manipulation tasks.

Several recent review papers have provided important perspectives on dexterous hands from specific thematic or application viewpoints. For example, recent reviews have examined dexterous hands in the context of intelligent manufacturing, with emphasis on structure, perception, grasping, manipulation skills, and manufacturing-oriented application scenarios [11]. Such work is highly valuable for clarifying the role of dexterous hands in industrial deployment. By contrast, the present review is not organized around a single-application domain. Instead, it aims to provide a system-level perspective that cuts across structure, perception, control, and deployment-oriented integration, while also considering broader drivers such as medical rehabilitation and human–robot collaboration. In this sense, the contribution of the present manuscript lies in clarifying the cross-layer coupling among embodiment, sensing, control, and deployability, rather than focusing only on task-oriented manufacturing applications.

Given their structural and functional resemblance to the human hand, anthropomorphic robotic hands have demonstrated strong potential across a variety of real-world scenarios. In industrial settings, anthropomorphic robotic hands are used for assembling intricate components and performing precise manipulation tasks. Beyond manufacturing, they also support service and medical applications, including household assistance, prosthetic reconstruction, and postoperative rehabilitation. In extreme scenarios such as disaster response or space exploration, they offer the potential to replace human hands in high-risk or inaccessible environments. Compared to traditional grippers or rigid manipulators, anthropomorphic robotic hands are better suited for collaborative scenarios involving physical interaction with humans. Their advantages include structural diversity, compliant control, and strong robustness to environmental uncertainty, making them ideal candidates for future human-centered robotic systems.

A particularly important trend within this broader development is the integration of soft robotics into dexterous hand systems. Soft materials, compliant actuators, stretchable sensors, and soft–rigid hybrid structures allow robotic hands to combine human-safe interaction with adaptive grasping and richer contact feedback. Recent soft prosthetic and soft robotic hand studies demonstrate that this trend is no longer limited to simple compliant gripping; instead, it increasingly supports tactile feedback, neuromorphic sensing, high-DOF prosthetic dexterity, and delicate in-hand manipulation [12–17]. Therefore, soft robotics integration provides a crucial bridge between structural embodiment, multimodal perception, and control, and it is treated in this review as a central direction for future dexterous hand development rather than as a peripheral material choice.

**Fig. 1.**
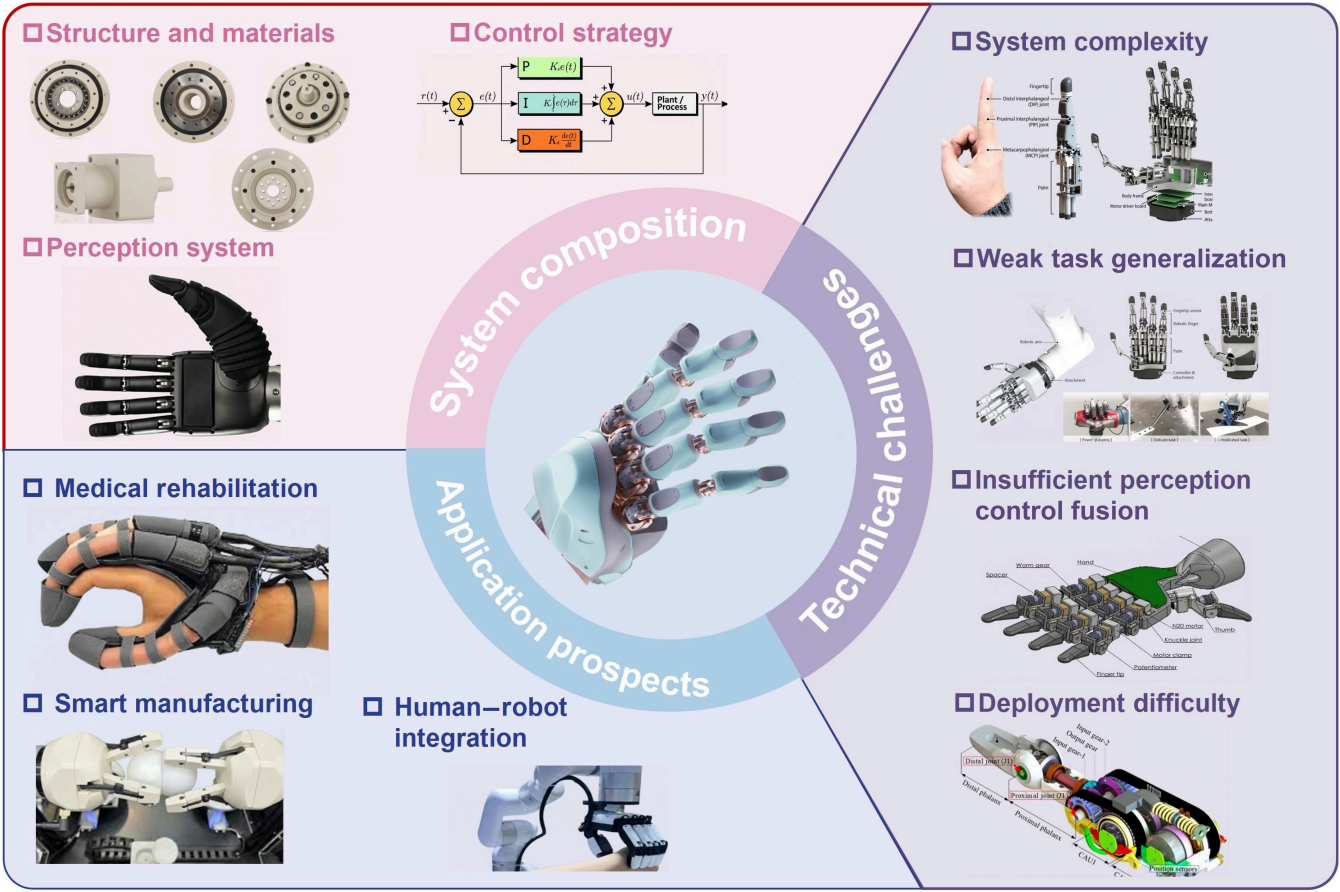
Visual summary of the technical framework proposed in this review, showing the relationship among system composition, technical challenges, and application-driven requirements in dexterous hand development [1,2,113,114]. Reprinted from [1,2,113,114].

To support the continued advancement and deployment of these systems, this review does not simply catalog recent developments in anthropomorphic robotic hands. Instead, it organizes dexterous hand technologies through a system-level technical framework consisting of 4 tightly coupled dimensions: structural embodiment, multimodal perception, control, and deployment-oriented integration. Figure 1 provides a visual summary of this technical framework by linking system composition, technical challenges, and application drivers, and it serves as the conceptual map for the remainder of the review. Structural embodiment includes morphology, actuation topology, compliance, and material-fabrication strategies, which determine the physical capabilities and constraints of the hand. Multimodal perception includes proprioceptive, tactile, and vision-based sensing together with their compact integration and fusion, which determine the observability and feedback quality available during manipulation. Control includes model-based, data-driven, and hybrid strategies, which translate embodiment and sensory information into dexterous behavior. Deployment-oriented integration includes modular architecture, communication interfaces, and hardware–software coordination, which determine whether the system can be efficiently maintained, scaled, and translated into real-world applications.

The key point of this framework is that these 4 dimensions are not independent. In dexterous hands, structural design directly affects sensor placement, observable information, and actuation bandwidth. Perception shapes the quality, latency, and interpretability of feedback signals available to the controller. Control strategies in turn impose requirements on sensing fidelity, computational resources, and actuator responsiveness. At the same time, deployment constraints such as interface heterogeneity, packaging, and communication architecture feed back into all 3 preceding dimensions under practical operating conditions. The framework proposed in this paper therefore organizes the field around these cross-layer dependencies, rather than treating structure, sensing, control, and deployment as isolated topics or discussing dexterous hands primarily from a single-application perspective.

While these foundational components provide the basis for human-level dexterity, several open challenges remain in practical implementation. To ground the discussion in practical contexts, typical examples are reviewed to identify key challenges. These include system complexity, limited task generalization, and the integration of sensing and control. Addressing these issues is essential for advancing real-world deployment. The review also examines how application scenarios such as intelligent manufacturing, medical rehabilitation, and human–robot collaboration actively drive dexterous hand development. Rather than treating these areas only as future application prospects, we use them as representative domains that impose distinct system-level requirements. Intelligent manufacturing emphasizes repeatability, cycle-time compatibility, maintenance efficiency, and robustness to part variability in addition to force and positional accuracy. Medical rehabilitation places greater emphasis on compliance, comfort, long-term safety, personalization, and user trust during continuous physical interaction. Human–robot collaboration requires not only safe contact behavior but also predictable responses, multimodal intent understanding, and reliable task adaptation in unstructured environments. These application-driven requirements provide an important basis for the later discussion of structure, perception, control, and deployment and help define the system-level scope of the present review.

Overall, the main focus of this review is to clarify how dexterous hand technologies for complex manipulation evolve and interact across 4 tightly coupled dimensions: structural embodiment, multimodal perception, control, and deployment-oriented integration. The scope of the paper therefore extends beyond a descriptive summary of representative hand platforms or isolated technical modules. Instead, it emphasizes the system-level coupling among morphology, sensing, decision-making, and deployability, while using intelligent manufacturing, medical rehabilitation, and human–robot collaboration as representative application drivers rather than as separate review topics. In this sense, the distinctive feature of the present manuscript lies in its deployment-oriented analytical perspective, which connects technical development with system complexity, task generalization, and sensing–control integration as the key challenges for future dexterous hand systems.

## Development and Classification

**Fig. 2.**
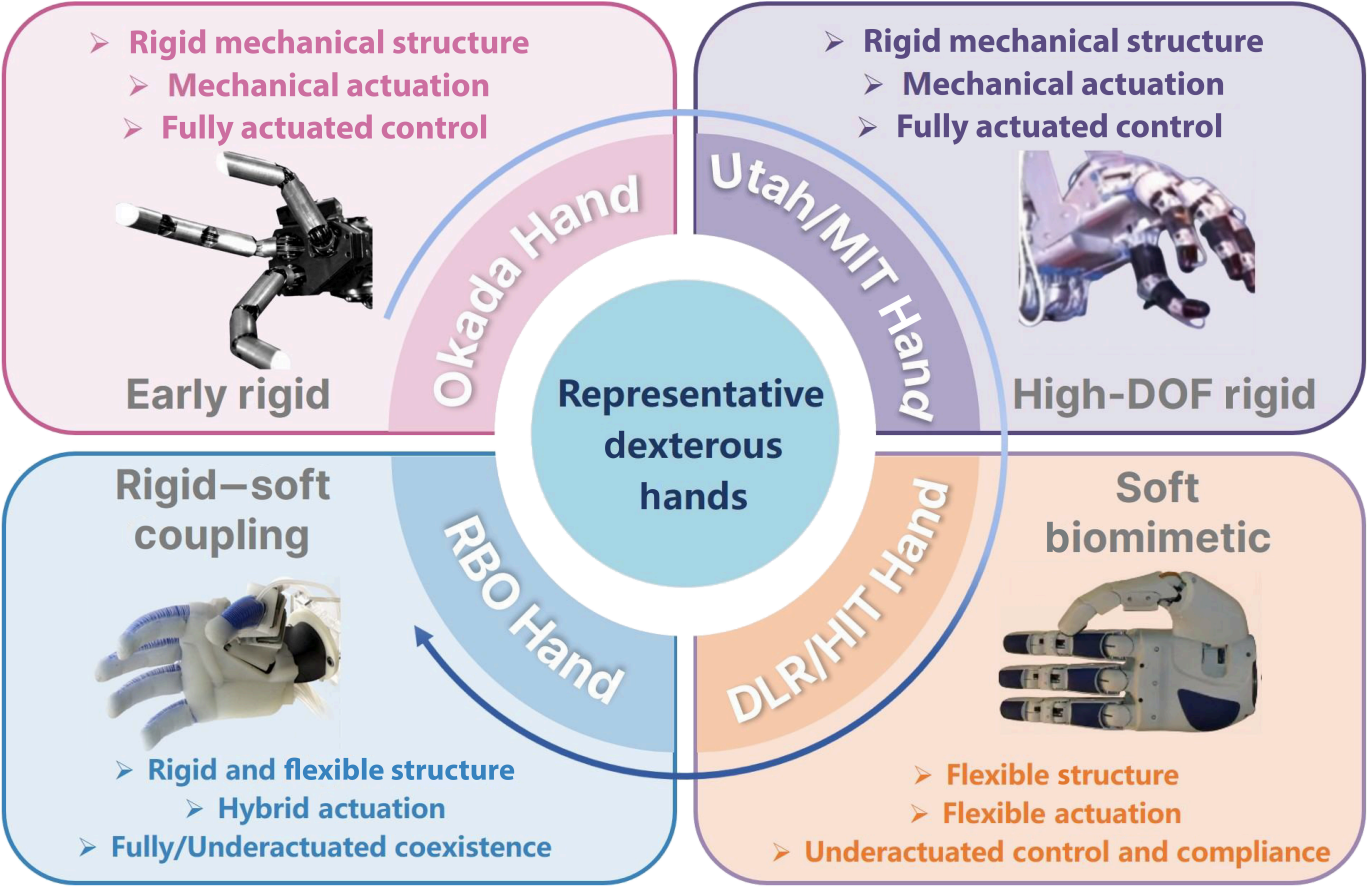
Intelligent evolution trends of dexterous hands [21,22,25,37]. Reprinted from [21,22,25,37].

The development of anthropomorphic robotic hands reflects a shift in robotics research from structural biomimicry to functional intelligence. This process follows a clear trajectory along 3 parallel technological lines: structure, materials, and control [18]. Early studies focused on multifingered rigid prototypes, serving as experimental platforms for basic grasping and actuation mechanisms. Over time, research expanded to include systematic modeling of human hand anatomy, material compliance, and control strategies. Substantial advances have been made in actuation methods and structural flexibility [19]. As a result, current systems exhibit diverse configurations and functional goals, showing strong potential for human-like grasping and dexterous manipulation. Figure 2 summarizes this developmental trajectory by illustrating how dexterous hands have evolved from early mechanically driven prototypes toward more intelligent, compliant, and integrated systems.

### Structural and design evolution

#### Rigid multifingered hands

Early research on anthropomorphic robotic hands focused on constructing rigid mechanical structures with multiple DOFs and contact points. These systems aimed to validate basic grasp modeling, force control, and finger coordination strategies. The Stanford Hand featured a 3-finger layout made of rigid materials and used cable-driven actuation to perform basic grasping [20]. It represented one of the earliest prototypes of multifinger mechanical hands. The Utah/MIT Hand further advanced the design by expanding finger arrangement and DOFs [21]. It included 4 fingers and 16 DOFs, each independently actuated through a cable system. This configuration enabled high-resolution motion control within a rigid frame and laid the foundation for future developments in structure–control integration and sensor feedback mechanisms. Although these systems differed from the human hand in form, they contributed valuable engineering experience in multifinger architecture, cable routing, and basic sensing integration. These efforts marked an important starting point for the evolution of dexterous robotic hands.

#### Soft biomimetic hands

With advances in materials science, compliant control, and bionics, the design of dexterous hands began shifting from rigid to flexible structures. Increasing emphasis was placed on faithfully replicating the anatomical structure and motion functions of the human hand [18]. The first stage of this transition was the development of high-DOF rigid biomimetic systems. Notable examples include the Shadow Dexterous Hand and DLR Hand II. These systems optimized joint configurations, finger coordination, and thumb opposition to enhance motion flexibility and grasp coverage [22–24]. Constructed primarily from metals or high-strength plastics, they used multiple independent actuators to drive each joint, achieving structural scales and DOF mappings more closely aligned with the human hand [19].

Soft robotics has reshaped dexterous hand design by shifting the emphasis from rigid kinematic replication to embodied compliance and adaptive interaction. Soft actuation mechanisms provide an important basis for this transition. Pneumatic actuators can generate large bending deformation and high compliance through pressure-driven chamber expansion, making them suitable for safe grasping and object-envelope adaptation. Tendon-driven compliant actuation combines flexible transmissions with soft or elastic joints, allowing underactuated fingers to conform passively to object geometry while retaining relatively compact actuation layouts. Dielectric elastomer and shape-memory-based actuators further expand the design space by enabling lightweight, electrically or thermally responsive actuation, although challenges remain in force output, response speed, durability, and control stability.

Soft–rigid hybrid designs have also become a major strategy in dexterous hand development. In these systems, rigid frames or skeletal components provide load-bearing capability and kinematic guidance, whereas soft skins, compliant joints, or flexible fingertips provide safe contact and adaptive deformation. This hybrid architecture helps balance 2 competing requirements: the need for compliance during physical interaction and the need for structural stability during force transmission and precise manipulation. However, soft–rigid integration also introduces challenges in material compatibility, interface durability, sensor embedding, and multiscale manufacturing.

Recent progress has also been made in fully soft dexterous hands, in which the fingers and palm are primarily composed of elastomeric or fabric-based structures. These systems offer strong adaptability to irregular objects and are particularly attractive for human–robot collaboration and assistive applications because of their intrinsic safety. At the same time, fully soft hands still face limitations in repeatable positioning, high-force manipulation, compact sensing integration, and long-term durability. These trade-offs indicate that soft robotics should not be viewed as a replacement for rigid dexterous hands, but as an expanding design paradigm that complements rigid and hybrid architectures.

Recent studies further show that the value of soft robotics integration lies not only in mechanical compliance but also in the cointegration of actuation, sensing, and feedback. Zhao et al. [12] developed an optoelectronically innervated soft prosthetic hand in which stretchable optical waveguides were embedded into soft fingers, showing how compliant structures can acquire distributed touch and curvature sensing while preserving safe deformation. Gu et al. [13] reported a soft neuroprosthetic hand that provides simultaneous myoelectric control and tactile feedback, demonstrating the importance of closing the loop between user intention, soft actuation, and sensory feedback in prosthetic applications. More recent prosthetic systems have also pursued human-level functional dexterity through lightweight multi-DOF architectures and compliant grasping mechanisms [14], while neuromorphic tactile sensing has been introduced to support precise and compliant grasping with biomimetic feedback pathways [15]. These examples indicate that soft robotic hands are evolving from passive compliant end-effectors toward perceptive and controllable human-interactive systems.

At the system level, soft robotics integration can be understood through 3 complementary routes. The first route is soft actuation, in which pneumatic, tendon-driven, dielectric, or thermally responsive structures generate motion through deformation and thereby reduce the burden of exact contact modeling. The second route is soft sensing, in which stretchable optical, capacitive, resistive, barometric, or neuromorphic tactile elements are embedded into deformable skins or fingers to provide contact, curvature, slip, and force information without compromising compliance. The third route is soft–rigid hybridization, in which rigid bones, links, or palm modules provide load-bearing and routing functions, while soft fingertips, skins, joints, or palms provide adaptive contact and safety. Zhou et al. [16] and Abondance et al. [17] respectively illustrate how soft robotic principles can be used to build anthropomorphic dexterity and delicate in-hand manipulation capability, suggesting that soft integration can support not only grasp stability but also manipulation richness. However, these advantages depend on solving persistent problems in deformation modeling, sensor calibration under large strain, actuator bandwidth, pressure or tendon routing, durability, and repeatable precision.

Representative examples include SoftHand Pro, FESTO BionicSoftHand, and the predominantly soft RBO Hand, all demonstrating strong performance in interaction safety and adaptability to unstructured environments [25,26]. More recent anthropomorphic hand platforms further enrich this development trajectory. For example, the CASIA hand represents a recent 15-DOF tendon-driven anthropomorphic robotic hand with human-like dimensions and dexterity [27,28]. By integrating the actuation system within the palm and combining anthropomorphic scale with tendon-driven transmission, it provides a representative example of how recent designs seek to balance structural complexity, compactness, and task-oriented dexterity. Related teleoperation-based studies on anthropomorphic hand–arm systems further demonstrate how such platforms can support human-like dexterous manipulation and coordinated hand–arm motion transfer. This evolution in structural form was paralleled by a shift in control strategies. The focus moved from precise trajectory tracking to compliance-based control, reflecting enhanced system tolerance to disturbances and greater adaptability to real-world variability. In addition to these platform-level examples, recent soft prosthetic and fully soft hand systems show that soft robotics integration is becoming a cross-cutting design principle across rehabilitation, prosthetics, and human–robot collaboration [12–17].

### Classification of anthropomorphic hands

#### Rigid and flexible structures

Rigid hands are typically constructed from metal, carbon fiber, or hard plastics. These materials provide high structural stability and enable precise pose control, which makes them suitable for industrial applications requiring accuracy and force execution. Representative systems include the DLR Hand and the Allegro Hand. In contrast, flexible hands are built using elastomers, fabrics, or composite materials that allow for deformation during contact. This adaptability enables them to conform to different object surfaces, offering improved collision tolerance and safety in human–robot interaction. Such flexibility is well demonstrated by predominantly soft systems such as the RBO Soft Hand and the Bionic series developed by FESTO. A more precise classification therefore requires considering where softness is introduced in the system: at the actuator, joint, fingertip, skin, palm, or sensing layer. This distinction is important because a hand may be mechanically rigid in its skeleton but soft at the contact interface, or predominantly soft in its fingers while still relying on rigid modules for actuation, pressure regulation, electronics, or attachment.

#### Mechanical and pneumatic actuation

Mechanical actuation typically employs servo motors, cable-driven systems, or linkage mechanisms. It offers fast response and high control accuracy, making it the predominant choice for rigid-structure hands. Representative systems include the Shadow Hand and the Robonaut Hand developed by NASA [29,30]. In contrast, pneumatic actuation is more common in soft robotic hands. These systems generate motion by pressurizing internal chambers, which deform elastomeric components and drive finger movement. This actuation method features a simple mechanical layout, high compliance, and favorable power-to-weight performance. However, it also poses challenges such as nonlinear control behavior and increased energy consumption. Notable examples include the RBO Soft Hand and the Bionic series developed by FESTO. Recent soft robotic hands also show that pneumatic actuation should be considered together with sensing and control integration: pressure regulation, chamber geometry, embedded strain or tactile sensing, and feedback strategy jointly determine whether soft fingers can move beyond envelope grasping toward dexterous manipulation.

#### Fully actuated and underactuated control

Fully actuated hands allocate a dedicated actuator to each DOF, enabling fine-grained posture control and the execution of complex gestures. This configuration is well suited for tasks that demand high dexterity. However, the increased number of actuators leads to greater mechanical complexity and a larger, heavier system. The DLR Hand II serves as a representative example. Underactuated designs address these limitations by reducing the actuator count through joint coupling or elastic linkages. Grasping is achieved via structural compliance, allowing the system to adapt passively to object contours. This approach simplifies the control architecture, lowers weight, and enhances both robustness and energy efficiency. Examples include the Yale OpenHand and the SoftHand Pro [26,31]. In addition to research-oriented open platforms, commercially accessible dexterous hands are also becoming increasingly important for evaluation and deployment. Recent work on the Inspire RH56DFX hand [32] shows that hardware characterization, analytical width-to-grasp planning, and hybrid closed-loop speed–force control can substantially improve the usability of a commercially available anthropomorphic hand for precision manipulation. This example is important because it links hand mechanism characteristics with deployable control methodology, rather than considering the platform only at the level of hardware configuration.

## Structural Design and Dexterous Motion

**Fig. 3.**
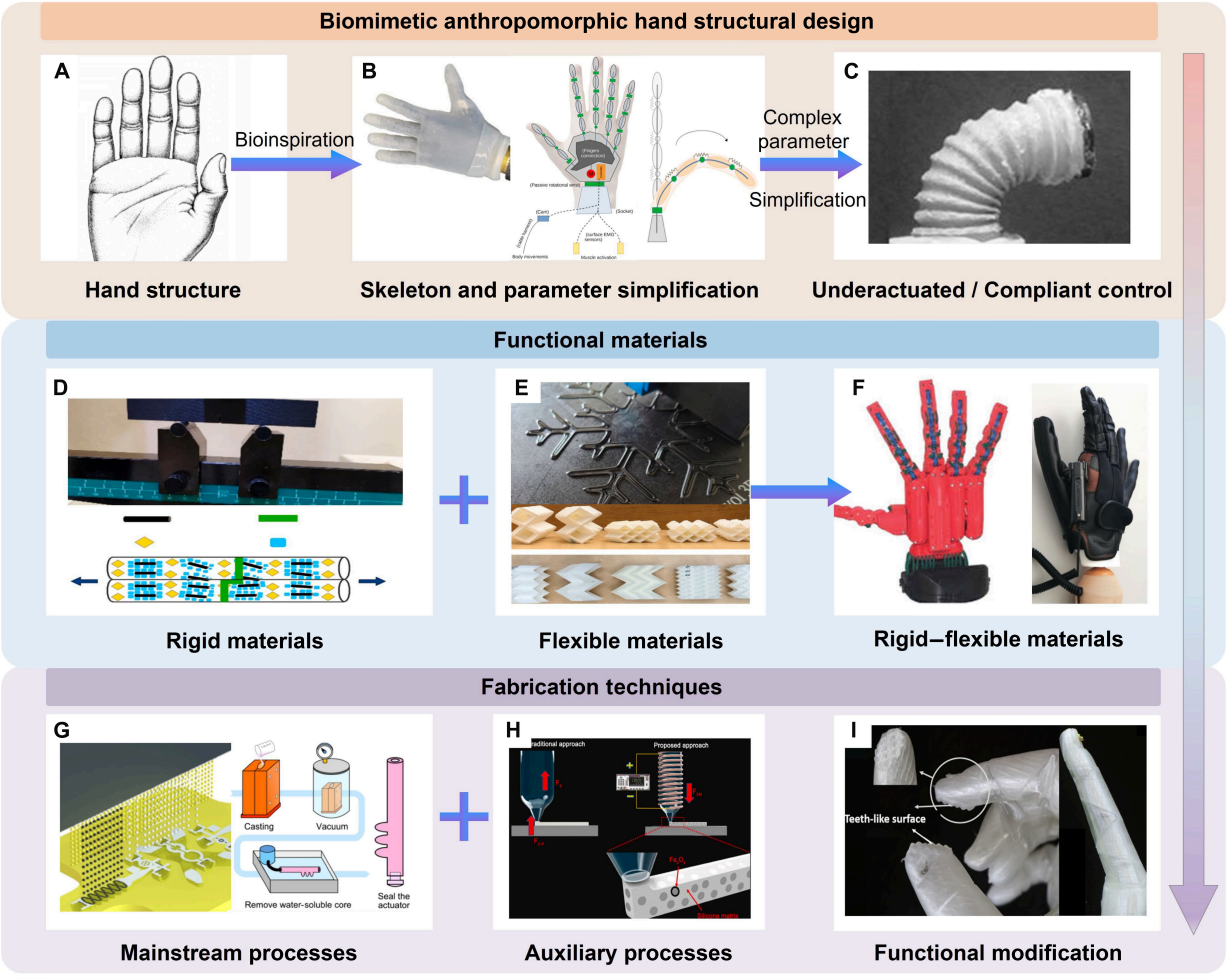
Structural design and fabrication of dexterous hands. (A) Human hand structure [115]. (B) Skeleton and parameter simplification [26]. (C) Underactuated/compliant control [44]. (D) Rigid materials [54]. (E) Flexible materials [45,48]. (F) Hybrid rigid–flexible materials [33,116].(G) Mainstream processes [56,57]. (H) Auxiliary processes [45]. (I) Functional modification [46]. Reprinted from [26,33, 44–46,48,54,56,57,115,116].

The development of anthropomorphic robotic hands has evolved from macroscopic biomimetic construction toward system-level optimization focused on functional realization. Against this developmental background, the following discussion focuses on the core strategies for achieving structural configuration and dexterous motion, including structural parameter design, underactuated and compliant control mechanisms, material selection, and advanced fabrication techniques. The goal is to develop structural platforms that are flexible, stable, and readily integrable. This is achieved through a combination of biomimetic abstraction, coordination of multiple DOFs, incorporation of compliant materials, and multimaterial fabrication. These foundations provide critical physical support for the integration of sensing modules and control strategies, offering a structural basis for unified perception–control systems. Figure 3 summarizes this structural embodiment layer by linking parameter design, compliant actuation, material selection, and fabrication strategies within a single development logic.

### Structural parameters

The human hand is a highly sophisticated anatomical system composed of multiple fingers, articulated joints, and coordinated control across a large number of DOFs. It serves as a primary reference in the structural design of anthropomorphic robotic hands [33,34]. To emulate human-like manipulation, robotic hands typically draw on abstracted parameters from the human hand, including the number of fingers, the segmentation of phalanges, the allocation of DOFs, and the configuration of thumb opposition. In a human hand, each finger apart from the thumb consists of 3 phalanges. Bending movements are achieved through the proximal interphalangeal and distal interphalangeal joints. The metacarpophalangeal joints support flexion and extension in one plane and abduction and adduction in another, enabling coordinated and refined finger movements. The thumb has a distinct kinematic structure that allows it to oppose the other fingers and typically includes 3 to 4 DOFs [35]. A standard human hand contains approximately 20 DOFs when the wrist is not considered. The wrist provides additional motion capabilities, including flexion, extension, radial deviation, ulnar deviation, pronation, and supination. When these movements are included, the total number of DOFs typically exceeds 23. As a result of this anatomical complexity, researchers have derived a series of structural design principles that guide the development of robotic hands. These principles seek to replicate key capabilities of the human hand within practical and scalable frameworks.

Inspired by the anatomical characteristics of the human hand, several design principles have emerged to guide the structural configuration of anthropomorphic robotic hands. One major objective is to retain key capabilities such as high DOFs, thumb opposition, and coordinated finger motion. At the same time, structural complexity must be adjusted to suit specific application needs [36]. Most designs follow the principle of a minimal functional set, aiming to preserve only the structural elements required for common manipulation tasks. Typical choices include a 5-finger layout, 2 or 3 phalanges per finger, and a 3-degree-of-freedom thumb. In configuring the DOFs, designers must strike a balance between dexterity, controllability, and system cost. Some systems prioritize biomechanical fidelity and flexibility, while others focus on practical feasibility and control simplicity [29,37]. These trade-offs have led to a variety of implementation strategies, which are exemplified in the structural configurations of representative robotic hands.

Different types of anthropomorphic robotic hands have adopted diverse structural configurations based on the design principles outlined above. The Shadow Hand closely replicates the human hand with a 5-finger, 3-joint structure and over 20 DOFs [29]. This allows for exceptional dexterity and manipulation capabilities. The Allegro Hand employs a 4-finger configuration, with 4 DOFs assigned to each finger [38]. It maintains a reasonable level of motion flexibility while optimizing structural compactness and control implementation. In contrast, the SoftHand Pro applies an underactuated approach [26]. Rather than assigning each joint an independent actuator, it relies on compliant materials and linkage mechanisms to achieve natural grasping behavior. This results in a mechanically streamlined architecture that trades full articulation for ease of implementation and control. These examples not only embody different structural configurations but also reveal how specific design choices shape the trade-offs between biological resemblance and system-level feasibility.

These systems illustrate different strategies for balancing structural complexity with biomimetic fidelity. Some prioritize functional replication of the human hand and are suited for tasks requiring high precision and versatility. Others emphasize design simplicity and control efficiency to improve usability and reduce cost, especially in routine grasping applications. The selection of structural parameters plays a critical role in defining the overall morphology of the hand. It also directly impacts motion behavior, control methods, and the integration of sensing modules. A well-structured mechanical foundation facilitates subsequent system integration by providing the necessary spatial arrangement and interface conditions.

### Underactuation and compliance-based control

Underactuated structures and compliance-based control mechanisms have been widely adopted in the development of anthropomorphic robotic hands to balance control complexity, structural compactness, and environmental adaptability. Underactuated designs reduce the number of actuators, thereby lowering system cost and simplifying control schemes. Compliance-based control leverages the material or structural flexibility of the hand, allowing it to conform naturally to target objects during interaction. This enhances grasp stability and operational safety. The integration of these 2 strategies provides a scalable, energy-efficient, and robust foundation for the construction of high-performance robotic hands.

#### Underactuated structural design

Underactuated designs refer to configurations in which the number of DOFs exceeds the number of actuators. These systems often achieve coordinated multijoint control through linkage mechanisms or elastic elements. A representative example is the Pisa/IIT SoftHand, which uses a single actuator to drive 19 joints [26]. Drawing on the biomechanical concept of motor synergies, it employs flexible tendon transmissions and compliant structures to realize natural grasping motions. While maintaining a high degree of biomimicry, this system substantially reduces control dimensionality and computational burden and has been widely adopted in rehabilitation support and human–robot interaction applications. Another example is the Yale OpenHand Model T, which combines modular and open-source design with elastic couplings and underactuated structures [31]. This approach balances ease of fabrication with practical grasping performance. The system supports 3-dimensional (3D) printing, facilitating the dissemination of underactuated strategies in education, research, and rapid prototyping contexts.

#### Compliant control mechanisms

Compliant control enhances adaptability to environmental variations through structural elasticity or material compliance, serving as a key strategy for handling unknown objects and operating in unstructured environments. The iHY Hand integrates flexible joints with an underactuated architecture, achieving stable and robust grasping through passive linkages [39]. This configuration effectively absorbs contact impacts and improves operational safety. Studies have shown that such structures can perform reliable grasps across various object types without the need for complex feedback mechanisms. The RBO Hand 2 employs pneumatic soft actuators to construct a compliant architecture [40]. By modulating internal air pressure, the system dynamically adjusts finger shapes, offering intrinsic compliance and enhanced enclosure capability during grasping. This design does not require precision joints or rigid components. Instead, it relies on material deformation to conform to diverse object geometries. This inherent compliance makes it particularly suitable for human–robot collaboration and tasks involving lightweight objects. Fully soft dexterous hands extend this idea by using deformable fingers and palms to perform gentle in-hand manipulation, where object repositioning emerges from coordinated compliance rather than only from rigid joint trajectories [17].

Beyond their individual advantages, these 2 strategies have increasingly been integrated into unified system architectures. The synergistic integration of underactuated structures and compliant control has been validated across multiple representative systems and has gradually emerged as a prevailing trend in dexterous hand design. This class of systems combines structural design, material selection, and control strategy to balance dexterity, adaptability, and resource efficiency [41]. Current research has moved beyond isolated implementations of underactuation or compliance, advancing toward their coordinated integration and system-level optimization. This evolution reflects a broader shift toward multiobjective design in dexterous manipulation. At the same time, the effectiveness of this strategy also depends on the mechanical compliance of high-performance materials and the structural compatibility afforded by manufacturing processes. The following section will analyze the material systems and selection principles that underpin these design strategies.

### Functional materials and mechanical properties

The application of underactuated structures and compliant control mechanisms imposes specific requirements on the mechanical performance of the materials involved. Flexible components must exhibit adequate compliance and stable deformation, while rigid elements are required to ensure structural support and fabrication accuracy. The selection of materials directly influences grasp stability and structural responsiveness and also affects the feasibility of coordinated multimaterial fabrication and system-level integration. This section reviews representative material classes used in dexterous hand systems, including elastomers, thermoplastic polymers, rigid structural materials, fabrics and textile-based components, metallic elements, and emerging soft functional materials. The purpose is not to provide an exhaustive catalog but to analyze how different material systems support compliance, load-bearing capability, sensing integration, and manufacturability in dexterous hand design.

In practice, material selection in dexterous hands is rarely determined by a single property. Instead, it reflects trade-offs among compliance, structural stability, fatigue resistance, interfacial compatibility, sensor embeddability, and fabrication accessibility. This is important for hybrid rigid–soft architectures, in which material combinations often determine not only local function but also the feasibility of system-level integration. For soft robotic hands, this trade-off is especially coupled with the sensing strategy: stretchable optical waveguides, soft capacitive skins, conductive elastomers, and neuromorphic tactile elements must maintain signal stability during large deformation while also preserving softness and wear resistance [12,15].

#### Silicone materials

Silicone is widely used as a core material in constructing flexible components for anthropomorphic robotic hands due to its excellent flowability, tunable mechanical properties, and biocompatibility. Represented by the Smooth-On Ecoflex series, silicone exhibits a Shore A hardness range typically from 00–10 to 00–50, allowing broad tunability of mechanical performance. For instance, Ecoflex 00-30 features an elastic modulus of approximately 0.1 to 1 MPa and a fracture elongation exceeding 500%, demonstrating high compliance and robust deformation capacity. These properties make it suitable for constructing multilayer flexible structures. The adjustability of these parameters provides essential support for realizing functions such as contact cushioning, shape adaptation, and safe object grasping.

Building on these properties, silicone has been widely applied in the casting of flexible structures and the integration of localized functional elements in dexterous robotic hands. Li et al. [42], through multimaterial integrated 3D printing, integrated Ecoflex silicone and Agilus material into the same structure, constructing the Tactile SoftHand-A system, achieving the monolithic integration of the flexible structure and the fingertip sensing layer, substantially enhancing tactile response capability and grasping compliance. To further expand silicone’s application in regional mechanical tuning, Manti et al. [43] employed silicones of varying hardness to construct a gripper with distinct stiff–soft partitions. Hard silicone was applied to enhance the stability of the dorsal finger region. In contrast, soft silicone was introduced at the fingertip to allow flexible deformation. This material distribution supported adaptive adjustment during grasping. In the STIFF-FLOP project, Cianchetti et al. [44] constructed soft modules using segmented-hardness silicone and integrated pneumatic actuation. The strategy combined structural compliance with controllable deformation patterns, highlighting the material flexibility and its effectiveness in complex actuation scenarios.

While the above applications highlight the functional versatility of silicone, achieving precise structural realization remains a key challenge in fabrication. To address structural instability during freeform silicone printing, Stano et al. [45] proposed an electromagnetically assisted extrusion strategy. By introducing electromagnetic perturbation during the extrusion process, the method effectively suppressed collapse phenomena and improved the print fidelity of thin-walled and hollow structures. This technique expanded the geometric boundaries of silicone-based structures and provided a new pathway for high-precision manufacturing of flexible components in dexterous robotic hands.

#### Thermoplastic polyurethane

Compared to liquid elastomers such as silicone that require mold-based fabrication, thermoplastic polyurethane (TPU) offers substantial advantages in direct manufacturability. It is compatible with mainstream fused deposition modeling (FDM) platforms and enables integrated 3D printing of soft and rigid structures. Mechanical evaluations indicate that widely available TPU materials, such as Ultimaker’s TPU 95A, can reach Shore A hardness levels of approximately 95. This hardness range supports the fabrication of hybrid structures combining flexibility and rigidity. Studies have reported elastic moduli ranging from 10 MPa to several tens of megapascals, with fracture elongation approaching 500%. In addition, TPU exhibits favorable fatigue resistance, allowing it to maintain stable deformation and structural integrity under moderate mechanical loads.

Building upon these characteristics, TPU-based flexible components have been incorporated into various dexterous hand systems. For example, Mohammadi et al. [46] proposed a prosthetic hand design framework based on 3D scanning and parametric modeling. Using TPU90 as a single flexible material to fabricate an integrated monolithic structure, the design ensured sufficient skeletal strength while forming a soft contact interface, effectively improving wearing comfort and joint impact buffering. In another study, Chahine et al. [47] developed a flexible pneumatic gripper whose finger modules were directly fabricated using TPU. The design featured internal locally bending air chambers, enabled controlled deformation under pressure, demonstrating TPU’s capacity to serve both structural and actuation roles in bioinspired fingers.

Although TPU demonstrates promising mechanical and processing characteristics, certain limitations emerge under high mechanical or cyclic loads [48,49]. Long-term durability may be affected by creep deformation and fatigue-related microcracks. Under high-cycle mechanical loads, TPU tends to develop microcracks and progressively fail. Inadequate bonding between printed layers can also reduce structural stability. Prior work has shown that TPU tends to form microcracks and degrade progressively under repeated loading. Moreover, the bonding strength between printed layers is critical to overall mechanical stability [50]. To address these issues, TPU is often employed as an intermediate flexible layer in combination with rigid skeletons or softer silicones. This approach enables the construction of systems that balance local compliance with global stability, meeting the demands of both shape adaptation and mechanical support [51].

#### Rigid materials

Rigid materials play a foundational role in the structural design of dexterous robotic hands. They are primarily used to construct internal skeletons, mounting platforms, and external support housings, and are also frequently employed as detachable molds in silicone casting processes. Common options include polylactic acid (PLA), carbon fiber-reinforced PLA (PLA+CF), and photopolymer resins. These materials offer high dimensional accuracy, mechanical rigidity, and good compatibility with 3D printing techniques, supporting the implementation of hybrid soft–rigid configurations.

Among the commonly used rigid materials, PLA stands out due to its ease of processing and consistent mechanical properties. It exhibits a tensile modulus of approximately 2 to 3 GPa, making it suitable for building structural frames on FDM platforms. Garcia-Samartín et al. [52] utilized FDM-fabricated PLA molds together with wax-assisted silicone casting to construct soft actuators containing internal cavities. This approach enabled repeatable fabrication of complex soft devices with reliable geometric accuracy.

While PLA is effective for general-purpose structural components, it is less suited to applications requiring finer resolution or microscale features. To complement PLA in such scenarios, photopolymer resins have emerged as an alternative. These materials are compatible with stereolithography and digital light processing platforms, which offer high resolution and excellent surface quality. Xavier et al. employed an elastic photopolymer resin to print a multichamber actuator using stereolithography [53]. The resulting structure achieved precise bending at each joint and demonstrated the feasibility of integrating soft and rigid elements at the microscale.

While PLA and photopolymer resins are well suited for general structural use, applications involving higher mechanical loads demand materials with enhanced strength and durability. In this context, PLA+CF offers superior stiffness and improved fracture resistance. Ogaili et al. [54] systematically evaluated short PLA+CF produced by FDM and reported substantial enhancements in stiffness and fatigue life compared to pure PLA. These results support its application in high-strength modules within dexterous hand systems. In addition, PLA has proven valuable as a mold material in complex structural fabrication. In the STIFF-FLOP project, detachable PLA molds were used in combination with silicone casting to construct segmented pneumatic modules [55]. This demonstrated the synergy between rigid mold frameworks and flexible components, offering a rapid and effective route for building soft robotic structures with complex geometries.

#### Other material systems and hybrid structural components

Beyond silicone, TPU, and rigid printed polymers, dexterous hand systems also employ a broader range of material systems depending on functional requirements. Fabrics and textile-based components are commonly used in tendon routing, compliant exoskeleton structures, and wearable assistive hands because they provide low weight, flexibility, and ease of integration with soft actuation. Metallic components remain important in load-bearing skeletons, transmission mechanisms, and precision joints, especially in systems requiring high stiffness, dimensional stability, and repeatable kinematic behavior. Hydrogels and other soft functional materials have also attracted interest in sensing and bioinspired interfaces due to their high compliance and responsiveness, although their durability and packaging remain challenging. In practice, many dexterous hands rely not on a single material class but on hybrid material systems that combine soft surfaces, compliant intermediate layers, and rigid load-bearing frameworks.

From a system perspective, 2 challenges are especially critical in material design for dexterous hands. The first is material compatibility. In hybrid rigid–soft systems, poor adhesion, modulus mismatch, differential curing or thermal expansion, and surface chemistry incompatibility can lead to interfacial failure, delamination, and long-term instability, particularly when silicones are integrated with rigid polymers, metals, or embedded sensors. The second is multiscale structural integration. Many dexterous hand designs require macroscale load-bearing frameworks together with microscale or mesoscale features such as fluidic channels, conductive traces, tactile microstructures, or embedded sensing units. Integrating such features across scales remains difficult because improvements in local functionality often come at the expense of fabrication complexity, structural robustness, or manufacturing repeatability.

### Fabrication techniques and construction methods

As anthropomorphic robotic hands evolve toward higher integration and compliance, their fabrication approaches have shifted from single-material printing to multimaterial coordination and multistage hybrid processing. Fabrication in dexterous hand systems increasingly relies on multiple coordinated routes rather than a single process. These include additive manufacturing, mold-based casting, machining-assisted fabrication, hybrid manufacturing, postprocessing for functional integration, and manual assembly. Overall, the central difficulty in fabricating dexterous hand systems lies not in the use of any single material or process but in achieving reliable integration across incompatible materials, structural scales, and functional subsystems.

#### Multimaterial cooperative printing

Multimaterial collaborative printing provides an effective pathway for the integrated realization of structural, actuation, and sensing components in dexterous robotic hands. Technologies such as PolyJet and dual-nozzle FDM allow the simultaneous deposition of rigid and flexible materials, enabling the direct formation of functional units in a single manufacturing step. This approach not only simplifies the assembly process but also enhances overall system reliability.

Such capabilities have been exemplified in recent research on fully integrated soft robotic systems. Hubbard et al. [56], based on the PolyJet platform, developed a fully 3D-printed soft robot that integrated a rigid skeletal frame, flexible pneumatic actuators, and fluidic logic control units. This design exemplified the potential of multimaterial printing for achieving tightly coupled functionality across mechanical and control subsystems. Beyond PolyJet-based systems, other studies have adopted alternative fabrication strategies to achieve similar integration. Despite its design flexibility, this printing approach still faces challenges in material compatibility, toolpath planning, and printing accuracy. In particular, the fabrication of microscale channels or the integration of precision sensors often requires additional postprocessing and coordinated material strategies for improvement.

#### Mold-based casting process

Mold-based casting remains a widely adopted fabrication strategy for dexterous hands and their soft structural components. It is particularly suitable for creating internal hollow chambers, flexible actuation units, and complex encapsulation structures. The process typically starts with molding using rigid materials like PLA or photopolymer resin, into which liquid elastomers such as Ecoflex are cast. After curing, a flexible component with precise geometry is obtained. In recent years, various innovative molding approaches have been proposed to enhance cavity precision and manufacturing efficiency.

Sacrificial-core strategies, including wax-based approaches, are valuable not because the underlying casting concept is new but because they illustrate how internal cavities and enclosed fluidic pathways can be realized within soft robotic structures [52]. In dexterous hands, the importance of such methods lies in their ability to support cavity formation and internal architecture control, although their usefulness is still constrained by mold accuracy, demolding complexity, and compatibility with larger hybrid structures. Similarly, Silva et al. proposed a single-step casting strategy using soluble cores, which avoids multiple mold disassembly and postprocessing steps and is particularly effective for the fabrication of pneumatic chamber actuators, thereby substantially improving manufacturing efficiency and structural integrity [57].

While these strategies have improved cavity precision and integration, mold-based casting still offers distinct advantages over direct 3D printing. It excels at replicating complex geometries, maintaining cavity integrity, and supporting flexible materials. These features are particularly important for regions in dexterous hands requiring compliance or internal voids. Nonetheless, the process remains multistepped and sensitive to mold accuracy and interfacial treatment between cores and elastomers. To further enhance its applicability and scalability, integration with advanced additive manufacturing and multimaterial design strategies is essential.

#### Electromagnetic and thermal-assisted printing

Flexible material printing often faces challenges including limited dimensional accuracy, structural sagging, and inadequate interlayer adhesion. To overcome these issues, researchers have introduced auxiliary techniques based on external physical fields, including electromagnetic stimulation and thermal treatment. These methods help improve the precision and structural stability of printed flexible components.

Stano et al. [45] developed an electromagnetic-assisted printing method by adding magnetic nanoparticles to silicone and applying an oscillating electromagnetic field during extrusion. This reduced flow resistance and improved the precision and stability of thin-walled structures. Without molds, the method enabled direct fabrication of complex hollow geometries. It is particularly suitable for rapid manufacturing of microstructured flexible units, such as multichamber and corrugated components in dexterous hands. In addition, Gu et al. [58] applied a 180 °C thermal treatment to TPU components printed by FDM. The treatment doubled the tensile strength and increased the elongation at break by over 70%. It also substantially improved interlayer adhesion and structural densification. This postprocessing method offers a practical means to enhance the fatigue life and mechanical reliability of flexible joints, with strong potential for engineering use. These auxiliary techniques complement conventional fabrication approaches, improving the feasibility of producing complex and integrated flexible systems in dexterous robotic hands.

#### Multiscale manufacturing and structural integration

A particularly important challenge in dexterous hand construction is multiscale manufacturing. Functional dexterous hands often require the simultaneous realization of macroscale structural frames, mesoscale compliant joints, and microscale features such as fluidic channels, tactile textures, conductive pathways, or embedded sensing interfaces. Although additive manufacturing and casting have improved geometric freedom, integrating these features across scales remains difficult. In particular, the embedding of microchannels or sensing units within larger load-bearing structures often introduces trade-offs among print resolution, material integrity, assembly complexity, and manufacturing yield. As a result, multiscale integration remains a major bottleneck for the transition from laboratory prototypes to robust and scalable dexterous hand systems.

#### Surface functionality and bioinspired construction

To enhance the surface functionality of dexterous robotic hands, some studies have introduced functional coatings and bioinspired structures after the primary fabrication process. For tactile sensing, Bounakoff et al. [59] encapsulated multiwalled carbon nanotube films within a flexible matrix, constructing an artificial rapidly adapting mechanoreceptor. This sensor uses percolation networks and nonlinear contact mechanics to preferentially respond to changes in pressure, exhibiting phasic sensing characteristics similar to biological receptors, thus providing robots with dynamic tactile perception capabilities. To improve grip performance, Hao et al. [60] designed fingerprint-inspired structures (such as whorl, loop, and arch patterns) on the surface of flexible robotic fingers. Experimental results showed that under lubricated conditions such as oil and water, these micron-scale groove structures effectively expel the lubricating medium, increase actual contact area, and substantially enhance the friction coefficient and gripping performance. In terms of environmental adaptability, Wang et al. [61] constructed superhydrophobic microstructured coatings on the gripper surface via a template method. This coating not only resists liquid wetting by forming an air cushion layer but also effectively prevents adhesion of dust, contaminants, and bacteria.

While not directly involved in structural fabrication, these techniques substantially enhance tactile response, gripping performance, and environmental adaptability. They further facilitate the construction of bioinspired skin and intelligent system integration.

The choice of fabrication strategy affects both the structural integrity and functional integration of dexterous hands, as well as the embedding of sensors and control systems. This is especially true for force and tactile sensors, where integration is closely linked to the manufacturing method. The relationship between fabrication and system integration will be further discussed in the following chapters, with a focus on sensory fusion and compliant control strategies.

## Perceptual Architecture and System Integration

**Fig. 4.**
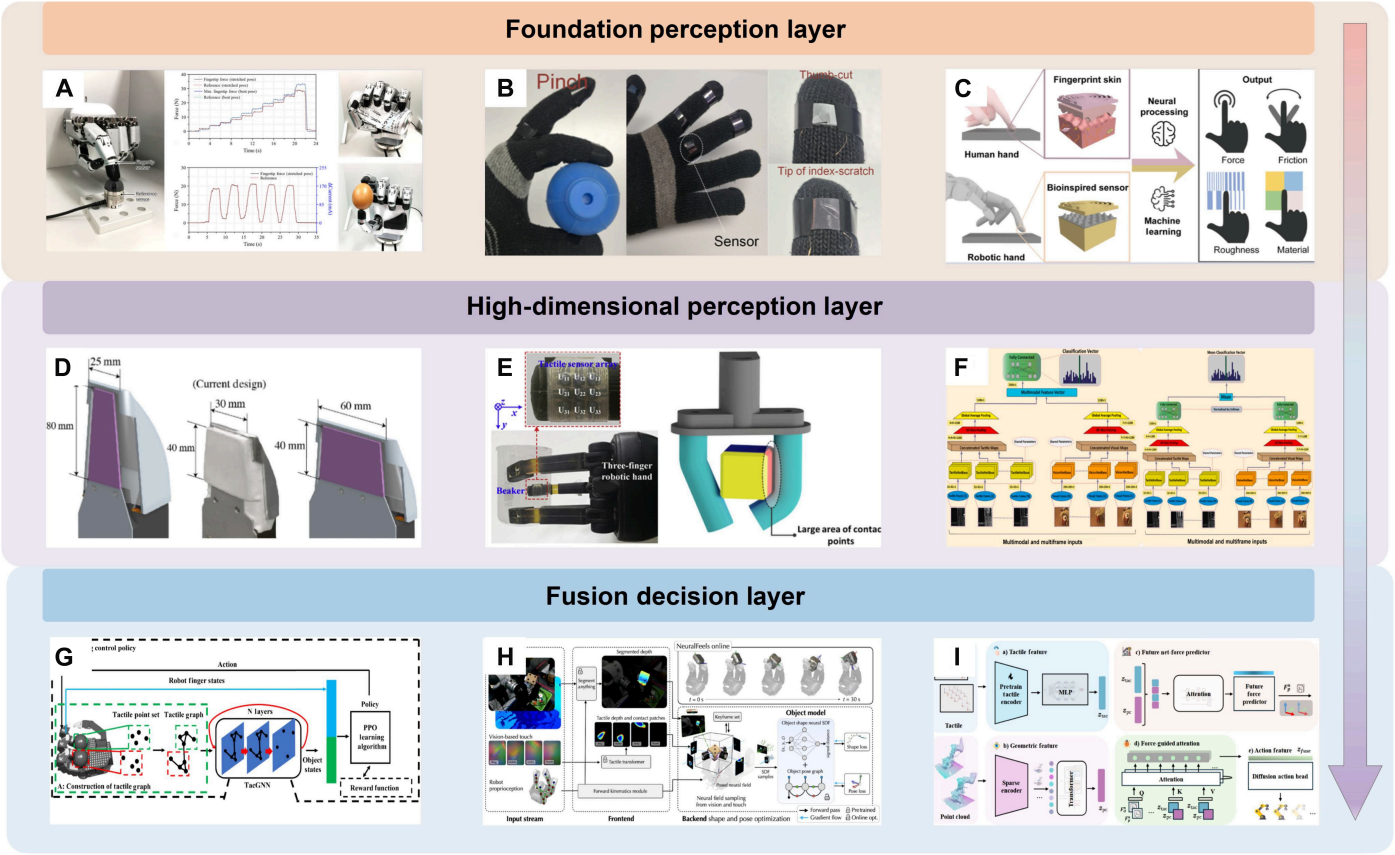
Multimodal perception system. (A) Joint angle and strain sensing [62]. (B) Tactile and pressure sensing [67]. (C) Friction and slip sensing [74]. (D) High-resolution contact geometry reconstruction [117]. (E) Force field estimation and modeling [68,118]. (F) Compact fusion perception modules [79]. (G) Tactile–proprioceptive joint modeling [80]. (H) Implicit multimodal modeling [83]. (I) End-to-end fusion strategies [84]. Reprinted from [62,67,68,74,79,80,83,84,117,118].

The sensory system of a dexterous robotic hand serves as the primary channel for acquiring both internal-state information and external-contact/environmental information and thus provides essential support for precise control and complex task execution. Unlike the structural and actuation pathways discussed in earlier sections, this section focuses on the functional organization and integration of sensory modules. From a system perspective, the perception architecture of dexterous hands can be divided into 2 complementary layers: proprioception, which captures the internal state of the hand such as joint angle, strain, bending, and finger configuration; and exteroception, which captures information about contact and the external environment, including tactile, pressure, slip, and vision-related cues. Building on this distinction, this chapter first reviews proprioceptive sensing technologies, then discusses exteroceptive sensing, and finally examines how these 2 streams are integrated through multimodal fusion to support dynamic grasping, in-hand manipulation, and task execution in unstructured environments. Particular attention is given to the construction of closed-loop frameworks that tightly couple perception and control. Figure 4 organizes these sensing modalities and fusion strategies into a unified perception architecture, illustrating how local sensing mechanisms connect to system-level multimodal integration.

### Joint angle and strain sensing

Joint angle and strain sensing constitute the core proprioceptive layer of the perception system in dexterous robotic hands. These modules provide information about the internal state of the hand, including finger posture, joint motion, structural deformation, and configuration-dependent actuation response, thereby supporting multi-degree-of-freedom control and closed-loop feedback.

#### Encoder-based angle sensing

In dexterous robotic hands with rigid structures, magnetic and rotary encoders remain the dominant solutions for joint angle sensing. Kim et al. [62] proposed the ILDA Hand, which adopts a linkage and ballscrew transmission mechanism and integrates Maxon ENX 8 magnetic encoders at the motor side. Through kinematic relationships, the system achieves 15 active DOFs (20 joints) for angle estimation. In addition, 6-axis force/torque sensors are installed at the fingertips to provide tactile feedback, enabling the system to maintain stable performance in closed-loop control for a variety of grasping tasks and tool operations.

#### Flexible strain sensors

Flexible strain sensors provide stretchability and strong surface conformity, which makes them particularly suitable for integration into soft robotic hands or hybrid rigid–flexible structures. Leveraging these properties, Attaoui et al. [63] developed the Fil3D-Hand system by embedding nanocomposite-based strain sensors at the joint regions. These sensors enable indirect estimation of finger bending through strain variation, supporting a fully integrated design that spans from low-cost additive manufacturing to full-range motion sensing. Experimental evaluations confirm that the system delivers stable and responsive posture feedback, even under high-speed dynamic grasping scenarios.

#### Optical fiber and waveguide-based sensing structures

In recent years, optical fiber-based sensing technologies have been increasingly adopted for joint angle detection in dexterous robotic hands, offering a promising alternative to traditional electronic sensors. As an early example, Capp et al. [64] proposed a design in which plastic optical fibers and readout electronics were monolithically integrated into multimaterial 3D-printed flexible fingers. This configuration enables self-sensing of finger posture without compromising compliance and achieves an average root mean square bending angle error of approximately 4.81°, while maintaining good adaptability to large deformations. Building upon this foundation, Amorebieta et al. [65] developed a multicore optical fiber vector bending sensor capable of detecting both bending magnitude and direction over a full 0° to 360° range, with high sensitivity to small angles (<1°), making it suitable for multidirectional posture measurement scenarios. Extending the concept further, Zhong et al. [66] introduced an uncalibrated dual-color-layer structure soft optical waveguide bending sensor, which can directly output bidirectional bending angles and directions with a resolution of 2.5°, requires no per-joint calibration under different curvature radii, and demonstrates robustness against shock and pressure disturbances, making it well suited for dynamic operation in soft robotic hands [66].

### Tactile and pressure sensing

Tactile and pressure sensing constitute a major part of the exteroceptive layer in dexterous robotic hands. Unlike proprioceptive sensing, which reflects the internal state of the hand, these modalities capture contact conditions at the hand–object interface and provide the external feedback needed for stable grasping, interaction monitoring, and environment-aware manipulation. These sensing modalities are primarily used to measure normal and shear force distributions across contact surfaces, identify pressure centers, and track temporal changes in contact force. Current mainstream approaches fall into 3 categories: microstructure-enhanced flexible capacitive sensors, distributed tactile sensor arrays, and low-cost multiaxis pressure sensing modules.

#### Microstructure-enhanced capacitive sensors

Capacitive sensors offer high sensitivity and strong adaptability to flexible structures. In recent years, their performance has been substantially improved through the incorporation of microstructural design. Luo et al. [67] developed a flexible capacitive pressure sensor integrated with an array of inclined micropillars. The sensor achieved a sensitivity of up to 1.48 kPa^−1^ within a range of 0 to 300 kPa, along with a response time of less than 1 s and excellent mechanical durability. These features make it well suited for localized contact force sensing in robotic hands.

#### Distributed flexible tactile arrays

To enable multipoint pressure sensing across entire fingers or palms, capacitive sensor arrays have been widely adopted. Shan et al. [68] developed a flexible tactile sensing array based on a multilayer structure incorporating an interleaved dielectric film. The design implements a 3×3 capacitive grid layout capable of detecting distributed pressure and center offset. It also supports the identification of sliding trends, providing a structural interface for subsequent friction estimation and control. In a parallel effort, He et al. [69] introduced a low-cost, large-area flexible capacitive array sensor fabricated using silver nanowire conductive films and polydimethylsiloxane dielectric materials. The sensor maintains linear response characteristics across a pressure range of 5 to 500 kPa and is well suited for wide-area force feedback applications in soft robotic grasping.

#### Multiaxis tactile sensing modules

Beyond single-axis pressure detection, some flexible sensing systems have been designed to provide multi-degree-of-freedom tactile feedback. Nugraha et al. [70] proposed a soft capacitive sensor with a single common dual-capacitor structure, capable of simultaneously detecting stretching, bending, and torsion within a single sensing unit. This design is suitable for integration into soft robotic fingers and actuator joints, providing multidimensional tactile feedback. In addition, vision-based tactile sensors have demonstrated great potential in multiaxis tactile measurement by providing rich contact information through optical sensing mechanisms. For example, the 9DTact sensor utilizes the optical and deformable properties of semitransparent gel to extract dense deformation information from raw tactile images without the need for additional markers or patterns [71]. These systems should be distinguished from vision–tactile perception frameworks, which combine separate visual and tactile information streams at the system level [71].

### Friction and slip sensing

Friction and slip sensing mechanisms are critical components for ensuring stable grasping and responsive contact control in dexterous robotic hands. These mechanisms are primarily used to detect slip onset, estimate frictional states, and predict grasp instability. Slip detection typically relies on analyzing shear force variations, surface pressure perturbations, and dynamic changes in tactile imaging to construct robust decision logic. Based on sensing principles and signal processing methods, existing approaches can be categorized into 3 types: neuromorphic tactile array response mechanisms, entropy-driven slip trend estimation, and bioinspired stick–slip detection strategies.

#### Neuromorphic tactile array mechanisms

Neuromorphic tactile arrays mimic the fast response characteristics of biological skin under dynamic stimulation, making them well suited for microslip detection and contact state classification. Sankar et al. [15] developed a biomimetic prosthetic hand integrated with a synapse-inspired tactile array. By analyzing temporal variations in electrical signals, the system rapidly identifies slip events and substantially improves grasp adaptability and precision. Similarly, Kim and Hwang [72] introduced the BaroTac system, which incorporates a miniature pneumatic sensor array capable of simultaneously detecting normal and triaxial shear forces. The system demonstrates high sensitivity to initial slip during multifinger cooperative manipulation.

#### Entropy-driven slip trend estimation

Slip behavior is often accompanied by rapid disturbances in the shear force distribution across the contact region. To capture this phenomenon, Hu et al. [73] developed a slip sensing framework based on the entropy evolution of contact force field images. Using tactile images acquired through GelSight, the system computes local entropy values and their temporal derivatives to predict slip trends dynamically. This method demonstrates strong robustness and accuracy even on irregular surfaces. By avoiding fixed threshold settings, it can be integrated with vision–tactile fusion networks, making it well suited for complex grasping scenarios.

#### Stick–slip feature recognition strategies

Stick–slip cycles during slip progression can serve as early indicators of grasp instability. Li et al. [74] designed a bioinspired system that captures stick–slip alternation within short time intervals using flexible microstructured sensors. By analyzing slip frequency and amplitude, the system enables early-stage slip prediction. Building on this idea, Zhao et al. [75] proposed a shear-to-normal force ratio model for estimating the instantaneous friction coefficient. The model integrates shear-to-normal force ratios with machine learning techniques to achieve precise slip prediction in flexible tactile hands.

### Vision–tactile perception systems

Vision–tactile perception refers to the fusion of separate visual and tactile information streams to improve object understanding, contact estimation, and manipulation decision-making. This concept should be distinguished from vision-based tactile sensing, in which tactile information is acquired through optical imaging mechanisms, as in GelSight-like sensors. In dexterous robotic hands, vision–tactile fusion is an important approach for enhancing interaction capability because it combines external scene information with local contact information.

#### High-resolution contact geometry reconstruction

High-resolution reconstruction of contact morphology is a key function of image-based tactile systems. The GelSight series exemplifies this approach by capturing fine deformations beneath a soft surface layer to reconstruct the 3D geometry of contact regions. Wang et al. [76] developed the GelSight Wedge, which combines an angled surface with a miniature camera to achieve submillimeter resolution [76]. This design enables detailed contact profiling for various end-effector tasks. The GelSlim system further introduced physical modeling and finite element inversion to support continuous force field estimation over time. Sodhi et al. later developed a method to map visual deformations to mechanical responses. This enables real-time estimation of local normal and shear forces during dynamic interactions, substantially improving the tactile responsiveness of soft robotic hands.

#### Force field estimation and modeling

To enable end-to-end estimation of contact forces from visual input, some methods reconstruct force distribution and normal/tangential components through regression or inverse modeling. Agarwal et al. [77] introduced a physically based rendering sensor design framework, which uses a large-scale dataset of synthetically generated tactile images from simulation to train deep neural networks. This method can robustly reconstruct contact geometry and force distribution even under sparse data and complex boundary conditions. It is well suited for challenging grasping and multiobject manipulation tasks. Swann et al. [78] proposed the Touch-GS method, a supervised learning framework that combines monocular depth estimation with tactile-guided implicit surface modeling to provide additional constraints for 3D Gaussian splatting. This method enables high-quality surface reconstruction of complex, reflective, or transparent objects. Compared to unimodal techniques, this multimodal strategy substantially improves both reconstruction accuracy and robustness, extending the applicability of vision–tactile reconstruction to broader scenarios.

#### Compact vision–tactile fusion modules

To meet the structural integration requirements of dexterous robotic hands, some studies have proposed miniaturized vision–tactile fusion modules that combine external visual information with tactile sensing in compact system architectures. Babadian et al. [79] developed the FusionNet series of neural architectures, which combine visual and tactile information using both early-fusion and late-fusion strategies for object recognition tasks. Studies have shown that, compared with unimodal approaches, multimodal fusion models achieve substantially better performance, demonstrating the great potential of multimodal fusion in high-dimensional information analysis. Overall, these advances push compact vision–tactile modules toward higher integration and intelligent fusion, supporting dexterous hands in complex manipulation scenarios. The vision–tactile fusion mechanisms described in this section not only enhance the ability of dexterous hands to perceive and interpret unstructured environments but also provide critical information for subsequent multimodal strategy learning and action decision-making, thereby further strengthening the synergy between sensing and control.

### Multimodal information fusion mechanisms

Dexterous hands must simultaneously integrate proprioceptive information (such as joint state and deformation) and exteroceptive information (such as contact, force, and environmental cues) in order to perform fine grasping, in-hand manipulation, and complex interaction tasks. Relying on a single sensing modality is insufficient for such dynamic and high-dimensional tasks. In response, recent work has developed system-level solutions that integrate multiple sensing modalities, including vision, touch, force, and proprioception. These systems often rely on end-to-end neural networks or implicit models to combine sensory data and improve control strategies. This integration substantially enhances decision-making and adaptability. Current research directions can be grouped into 3 categories: strategy learning based on tactile–proprioceptive fusion, implicit modeling frameworks for multimodal shape perception, and end-to-end execution systems incorporating attention mechanisms.

#### Tactile–proprioceptive joint modeling

Yang et al. develop a tactile graph neural network that maps tactile signals into graph-structured inputs while combining finger joint-state encoding [80]. This framework supports end-to-end policy learning for blind manipulation scenarios. It enables manipulation behavior prediction without visual input and shows strong generalization and adaptability in multiple-object in-hand rearrangement tasks. Unlike traditional tactile-driven approaches, the tactile graph neural network transforms low-dimensional tactile signals into learnable structured features, endowing policy learning with spatial semantic parsing ability.

#### Implicit multimodal representation

TouchSDF reconstructs tactile point cloud data from vision-based tactile images and embeds them into an implicit field using the DeepSDF framework. This approach generates high-resolution representations of contact surfaces, enabling continuous modeling of object geometry and supporting downstream contact planning [81]. Building on this concept, ViTaSCOPE introduces a shared feature space for integrating visual and tactile information, which allows simultaneous estimation of object pose, contact points, and grasp locations [82]. This method supports plug-and-play perception for previously unseen objects. NeuralFeels further extends the implicit modeling framework by encoding visual, tactile, and joint motion data into a unified neural field [83]. This enables a continuous semantic representation of interaction states and supports real-time modeling and adaptive feedback during dynamic in-hand manipulation.

#### End-to-end fusion strategies

To achieve adaptive integration of different sensory modalities during task execution, the Adaptive Fusion framework introduces predictive attention mechanisms that dynamically adjust the weights of visual, tactile, and force inputs [84]. A multibranch fusion network is used to represent these modalities hierarchically, which has been shown to outperform static fusion strategies in tasks such as grasp force control and stability estimation. In addition, GelFusion enhances robustness under visual occlusions by compensating for missing visual data with tactile feedback, while FingerSLAM improves 3D surface reconstruction accuracy through strengthened tactile–visual integration [85,86]. These improvements further expand the applicability of multimodal fusion systems. Mao et al. [87] proposed an integrated framework that combines visual inputs with thermally sensitive tactile signals. By employing a convolution–attention network, the system achieves complex object recognition and perception of operational intent and demonstrates excellent strategy generalization and task stability specifically in domestic service scenarios, thereby further confirming the scalability of multimodal fusion systems in practical environments. The methods outlined in this section represent a shift from perception-level integration toward tightly coupled perception–policy loops. These systems support adaptive understanding and feedback regulation, establishing a semantic and architectural foundation for the control strategies discussed in Control Strategies and Learning Methods.

## Control Strategies and Learning Methods

**Fig. 5.**
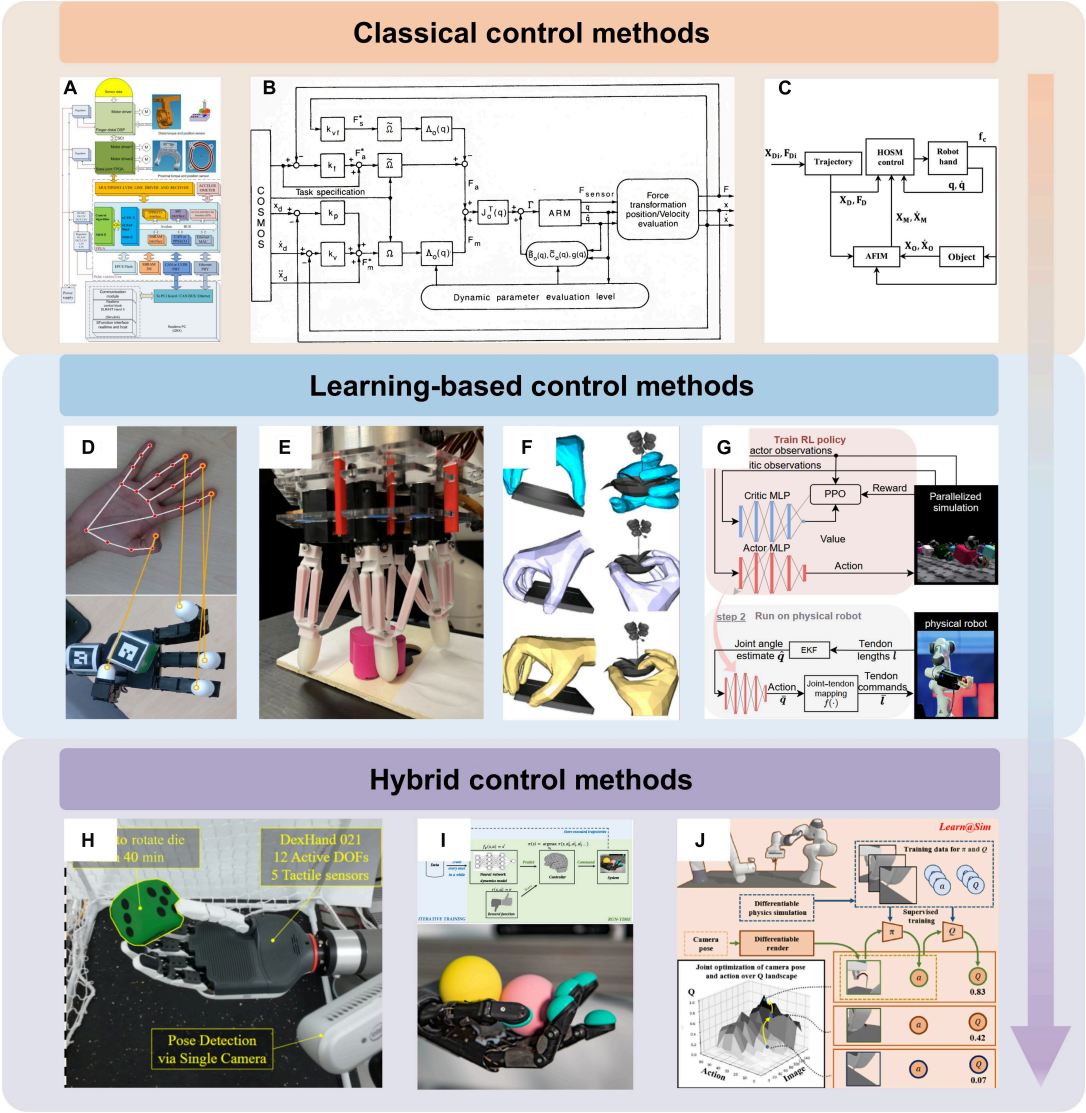
Control strategies and learning methods. (A) Impedance control [119]. (B) Operational space control [91]. (C) Hybrid force–position control [120]. (D) Dexterous imitation made easy [121]. (E) Imitation learning [94]. (F) Deep grasping models [122]. (G) Learning a dexterous policy for a biomimetic tendon-driven hand [123]. (H) Multigoal dexterous hand manipulation using probabilistic model-based reinforcement learning [124]. (I) Deep dynamics models for learning dexterous manipulation [99]. (J) Sensing-aware model-based reinforcement learning [102]. Reprinted from [91,94,99,102,119–124].

The development of control strategies has served as a central foundation in advancing human-like manipulation. Early methods were based on explicit modeling of kinematics and dynamics, aiming to establish stable and accurate motion control. In recent years, new paradigms have emerged that incorporate data-driven approaches and hybrid modeling techniques. These advances have led to control systems with improved adaptability, generalization, and the ability to integrate multimodal information. This chapter focuses on control strategies as the core of dexterous hand development. It follows the structural design and perception systems discussed in previous chapters and aims to clarify the key mechanisms and technical pathways for realizing dexterous manipulation. The insights presented here lay the theoretical groundwork for the system integration addressed in the next chapter. Figure 5 summarizes the major control paradigms discussed in the following section and highlights the transition from analytical control to learning-based and hybrid strategies.

### Classical control methods

In the early stages of dexterous hand development, researchers primarily relied on explicit modeling of system kinematics and dynamics to design control strategies. These methods are grounded in well-defined physical models and emphasize clarity in structural formulation and interpretability. Control laws are derived based on complete system models, aiming to achieve precise control over high-degree-of-freedom systems under conditions of accurate modeling. Representative strategies include impedance control, operational space control, and hybrid force–position control. These approaches have been widely applied to classical platforms such as the DLR-HIT II and the Barrett Hand, providing effective solutions for multijoint coordination and fine manipulation.

#### Impedance control

Impedance control was initially proposed by Hogan [88] with the goal of configuring robotic systems to exhibit specified dynamic behavior, characterized by mass, damping, and stiffness parameters. This method enables the system to respond compliantly during contact interactions and is particularly suitable for manipulation scenarios involving uncertain physical contact. In the domain of dexterous hand control, it has shown substantial applicability.

On the DLR-HIT II platform, researchers developed a Cartesian impedance control framework in task space, which integrates flexible joint modeling and friction compensation. This framework enables adaptive fingertip tracking under dynamic conditions. Experimental results demonstrate that adjusting impedance parameters allows the system to balance response speed and contact force. Further comparisons indicate that joint-space impedance control provides better robustness against friction disturbances, while Cartesian-space control achieves higher precision in trajectory tracking. The Barrett Hand platform, based on complete kinematic and dynamic modeling, serves as a foundation for integrating impedance control with multistrategy control architectures. Detailed studies of its modeling procedures and system characteristics provide a solid basis for advancing traditional model-based control approaches [89,90].

#### Operational space control

Operational space control was proposed by Khatib [91] to address the coupling and redundancy challenges in joint-space control. This method builds the control law directly in task space, which simplifies motion and force coordination for multi-degree-of-freedom systems. The approach transforms system dynamics using the Jacobian matrix and task-space inertia formulation, allowing clearer control structures and easier interpretation. In dexterous hand applications, this control strategy is often combined with impedance control to manage both trajectory accuracy and contact force. For example, in the DLR-HIT II platform, researchers developed a task-level controller with built-in compliance and stability features. This controller improves adaptability and robustness in complex manipulation tasks.

#### Hybrid force–position control

In many multidimensional manipulation tasks, certain directions require precise force regulation, while others demand accurate position tracking. Hybrid force–position control addresses this challenge by dividing the task space into 2 orthogonal components. One component handles force control, while the other manages motion. Independent control laws are applied to each, enabling coordinated regulation and allowing the system to adapt to complex and variable environments. Hasan et al. [89] developed a hybrid controller for the Barrett Hand by combining impedance modulation with sliding-mode control. This approach enables stable grasping and fine manipulation of target objects, even under nonideal contact conditions. Simulation results demonstrate fast response and strong disturbance rejection, confirming its feasibility in dynamic tasks.

Model-based control methods achieve high precision and stability in systems with clearly defined structures and accurate analytical models. Their strong physical basis and interpretability make them effective under ideal conditions. However, these methods rely heavily on precise modeling and often struggle with structural flexibility, uncertain contact conditions, and environmental variability. They also lack adaptability, require extensive parameter tuning, and provide limited support for multimodal sensing or online adjustment. These limitations have motivated a shift toward more flexible and adaptive strategies. In particular, data-driven and hybrid learning-based approaches offer promising solutions to improve generalization and autonomous optimization. The next section explores how learning-based methods enhance the adaptability of dexterous hands in unstructured environments and address the key limitations of traditional model-based control.

### Data-driven learning-based control methods

Traditional model-based control methods are becoming less effective as dexterous hand systems grow in complexity and are required to operate in increasingly dynamic environments. These methods depend on accurate analytical models, which makes them difficult to apply to systems with flexible structures, uncertain contact conditions, or external disturbances. They also struggle to handle diverse tasks and lack the adaptability needed in unstructured scenarios. To address these challenges, researchers have adopted data-driven strategies. By learning control policies from interaction data, these methods enable autonomous control without the need for explicit system modeling. Depending on the learning paradigm, common approaches include reinforcement learning, imitation learning, self-supervised learning, and combined imitation–reinforcement strategies that use demonstrations to initialize or regularize exploration. In addition, deep models trained on large-scale grasp datasets have shown growing utility in dexterous manipulation. The following sections present representative advances in each of these areas.

#### Reinforcement learning

Reinforcement learning (RL) optimizes control policies through interaction with the environment by using reward signals to guide learning. It enables end-to-end policy training and demonstrates strong adaptability to environmental variations. The OpenAI team employed a deep RL framework to train the Shadow Hand for complex in-hand object rotation and reorientation. By leveraging large-scale simulation, they accelerated policy convergence and successfully transferred the learned strategy to physical hardware [92]. Subsequent research introduced automatic domain randomization (ADR), which substantially enhanced the robustness of the policy under real-world disturbances. This improvement enabled the robot to achieve autonomous resolution of a 3×3 Rubik’s Cube [93].

#### Imitation learning

Imitation learning (IL) trains control policies using expert demonstrations, thereby avoiding the inefficient exploration process required in RL. This method offers high sample efficiency and rapid training. Researchers employed the TeleHand teleoperation platform to collect human demonstrations on the DeltaHand system [94]. To improve performance across tasks, they combined behavior cloning with policy fine-tuning, achieving high success rates in dexterous manipulation. In the context of soft robotic hands, other studies proposed object-centered demonstration frameworks to improve policy adaptation to compliant structures. Additional research modeled human grasp behaviors to develop generalized manipulation strategies, achieving human-like control capabilities across diverse tasks.

#### Combined imitation and reinforcement learning

In dexterous manipulation, imitation learning and RL are increasingly combined to leverage the strengths of both paradigms. Demonstrations can provide an informative initialization that reduces the exploration burden in high-dimensional action spaces, while RL can further refine the policy through interaction and improve robustness beyond the demonstrated trajectories. This combination is particularly valuable for dexterous hands, where sparse rewards, contact uncertainty, and high-dimensional control often make pure RL difficult to scale. In this sense, IL+RL offers a practical intermediate route between purely demonstration-driven learning and fully autonomous policy optimization, and has become an important strategy for improving sample efficiency and deployment feasibility in dexterous manipulation.

#### Self-supervised learning

Self-supervised learning (SSL) avoids reliance on externally labeled data by constructing a feedback loop based on system-generated sensory input and interaction results. This approach is well-suited to tasks with high labeling costs or complex environmental disturbances [95]. For example, 1 study combined RGB-D perception with tactile feedback to derive internal success signals. These signals enabled real-time policy updates and improved robustness, leading to substantial performance gains in dynamic grasping tasks.

#### Deep grasping models

Beyond the conventional learning paradigms, deep neural network models trained on large-scale grasp datasets have become an important direction in dexterous hand control research. The DexGrasp series developed extensive multifinger grasping datasets and used deep generative models guided by object features. This approach enabled the system to perform generalized grasping across a wide range of heterogeneous objects [96,97]. In another example, the GrainGrasp model introduced fine-grained contact prediction, improving the precision of motion planning and enhancing grasp stability during manipulation. More recently, large-scale human-to-robot transfer has emerged as a new direction in dexterous manipulation learning. A representative example is Sharpa Hand [98], which trains a vision–language–action model on 20,854 hours of action-labeled egocentric human video and demonstrates that large-scale human data can serve as a predictable supervision source for high-degree-of-freedom dexterous manipulation. The resulting policy improves average success rate by 54% over a no-pretraining baseline on a 22-DOF dexterous robotic hand and also transfers to robots with lower-DOF hands, suggesting that large-scale human motion can provide an embodiment-agnostic motor prior for dexterous hand learning. This line of work usefully extends dexterous hand research from platform-specific control toward scalable learning from human behavioral data.

### Hybrid control methods integrating modeling and learning

Model-based control methods offer clear physical interpretability but often lack adaptability in complex environments. In contrast, data-driven methods are more flexible but face limitations in training efficiency and generalization. To address these challenges, researchers have increasingly explored hybrid control strategies that combine explicit modeling with learning mechanisms. These approaches maintain the clarity of analytical models. At the same time, they introduce adaptability through data-driven mechanisms. This combination enables improved performance in unstructured and dynamic conditions. Representative strategies include model-based RL, residual RL, and perception-driven adaptive control architectures.

#### Model-based RL

Model-based RL improves training efficiency by constructing differentiable or probabilistic dynamics models that allow the control policy to update based on model predictions. These models allow the control policy to update based on model predictions, substantially reducing sample requirements and improving training stability. For example, Nagabandi et al. [99] used a neural network to model system dynamics and applied model predictive control, enabling the Shadow Hand to perform precise object manipulation with limited data [99].

#### World-model-based control

Beyond classical model-based reinforcement learning, recent work has increasingly emphasized world-model-based control, in which a compact predictive model of hand–object interaction dynamics is learned and used for planning, control refinement, or model-predictive decision-making. Compared with conventional dynamics modeling, world models place greater emphasis on latent-state prediction and long-horizon rollouts, making them attractive for dexterous manipulation tasks that involve delayed effects, contact uncertainty, and high-dimensional multimodal feedback. In dexterous hand systems, such approaches are promising because they can provide an internal predictive representation that supports more efficient policy adaptation and reduces the dependence on exhaustive real-world interaction. This direction therefore extends model-based learning from explicit dynamics fitting toward more general predictive control frameworks.

#### Residual RL

Residual RL enhances existing controllers by learning corrective policies on top of pretrained or analytical strategies. This method is particularly effective in compensating for modeling errors and external disturbances, offering both robustness and transferability. Davchev et al. [100] fused demonstration-based policies with residual RL modules, establishing a more stable policy update path. Ceola et al. proposed the RESPReCT framework, which substantially accelerated policy convergence in multifinger dexterous hand platforms [101].

#### Perception-driven reinforcement learning

Incorporating perception feedback into hybrid model-learning frameworks can further enhance system responsiveness in dynamic environments. Lv et al. [102] introduced the SAM-RL architecture, which combines tactile and visual signals to adjust control strategies based on real-time perceptual input. By addressing the limitations of static model parameters, this framework enables adaptive responses in complex and unstructured scenarios. As a result, the controller shows improved generalization across different tasks and under external disturbances.

Hybrid control approaches maintain a balance between accuracy, robustness, and adaptability. Their effectiveness relies on the seamless integration of control design and learning components, which supports efficient manipulation across multiple tasks and complex contact conditions. Future work will focus on strengthening the coupling between modeling and perception to enhance policy transferability and enable real-time deployment.

### Comparative analysis of control strategies

Rather than reintroducing individual control paradigms, this section compares model-based, data-driven, and hybrid strategies along several cross-cutting dimensions that are especially relevant to dexterous hand deployment, including interpretability, adaptability, sample efficiency, computational burden, hardware dependence, and real-time applicability. From this perspective, the key question is not which category is universally superior but how different control strategies align with different embodiment, sensing, and task conditions.

Model-based strategies remain advantageous when interpretability, stability, and precise control under well-characterized conditions are the dominant priorities. Their strength lies in the explicit use of kinematic and dynamic structure, which supports predictable behavior and principled controller design. However, their effectiveness decreases in the presence of compliant embodiment, uncertain contact, and task variability, where accurate modeling becomes difficult. Data-driven strategies, by contrast, offer greater flexibility in dealing with complex interaction dynamics and high-dimensional sensory input, and they are often better suited to tasks requiring adaptation across variable environments. Their main limitations are the dependence on large datasets, substantial computational cost, and sensitivity to transfer conditions between training and deployment. Hybrid strategies occupy an intermediate position by combining analytical priors with learning-based adaptation. Their main value lies not simply in blending 2 methodologies but in balancing physical consistency, robustness to uncertainty, and deployment feasibility when neither purely model-based nor purely data-driven solutions are sufficient.

From a deployment perspective, these trade-offs become even more substantial. Systems with stable rigid structures and relatively controlled environments may benefit more directly from model-based precision, whereas systems with compliant embodiment and rich multimodal feedback often require stronger adaptation capability. Hybrid strategies are especially attractive in dexterous hands because they can better accommodate the tight coupling among embodiment, sensing, and control while still retaining partial interpretability and data efficiency. In this sense, the purpose of this comparative section is not to restate the characteristics of each control category but to clarify how their relative advantages shift under different structural, perceptual, and deployment constraints.

## Deployment-Oriented Integration of Dexterous Hand Systems

**Fig. 6.**
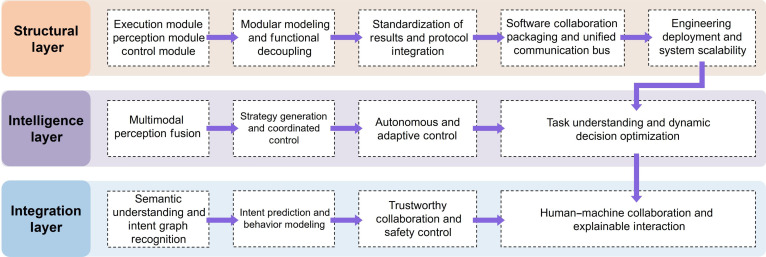
Deployment-oriented evolution framework of dexterous hand systems, illustrating how structure, perception, control, and integration move from isolated functional modules toward coordinated and deployable architectures.

Dexterous hand systems are gradually progressing from research prototypes to engineering applications. In this context, modular architectures have been explored as 1 possible route to improve maintainability, task reconfiguration, and system scalability. However, in dexterous hands, modularization is not universally beneficial. Because sensing, actuation, embodiment, and control are often tightly coupled, the separation of these functions into standardized modules may improve replaceability and development efficiency in some cases, while simultaneously introducing bandwidth bottlenecks, packaging overhead, reduced space efficiency, and constraints on system-level co-optimization in others. Figure 6 visualizes this transition from isolated functional advances toward coordinated and deployable dexterous hand architectures, and is used here to synthesize the integration-oriented perspective.

### Deployment-oriented modular design and standardized interfaces

Viewed through the technical framework proposed in this review, dexterous hand systems are evolving from isolated advances in structure, sensing, or control toward coordinated and deployable system architectures. Their structural design is also shifting from fixed functional layouts to modular architectures that support task reconfiguration and system scalability. Many multifingered hands already feature biomimetic designs and a certain degree of mechanical flexibility. However, they still struggle with adapting to varied tasks, responding to environmental changes, and ensuring efficient maintenance. To address these limitations, recent studies have explored modular architectures in which execution, actuation, and sensing units are separated and connected through defined interfaces. While such approaches can improve system flexibility and facilitate module replacement, their effectiveness depends strongly on the maturity and compatibility of the underlying subsystems. In dexterous hands, where sensing, actuation, and control are tightly coupled, modularization is not universally beneficial and must be evaluated in the context of system-level constraints. For example, the Yale OpenHand project emphasizes open-source design and fabrication accessibility, enabling rapid prototyping and dissemination of underactuated hand designs [103]. Rather than representing a system-level modular standard, its design highlights the importance of accessible hardware platforms for iterative development and experimental validation.

However, modularization and interface standardization introduce nontrivial trade-offs in dexterous hand systems. In particular, large-scale tactile sensing generates high-dimensional data streams with heterogeneous sampling rates, which can create bandwidth and latency bottlenecks when routed through standardized interfaces. Similarly, strict interface abstraction may reduce design flexibility, increase packaging complexity, and limit the co-optimization of structure, sensing, and control. These issues are especially pronounced in systems requiring high-frequency feedback and tightly coupled actuation loops. As a result, excessive modularization may degrade overall system performance if not carefully aligned with sensing and control requirements.

Alternative routes should also be considered when improving the performance, practicality, and deployability of dexterous hand systems. One important direction is codesign, in which embodiment, sensing, and control are optimized jointly for specific task requirements rather than being separated into loosely coupled modules. This approach is particularly valuable in compact hand systems where space efficiency, response speed, and local integration are more critical than module interchangeability. In addition, the scaling challenge associated with high-dimensional visual and tactile information may be addressed through data-efficient processing strategies such as neuromorphic computing, multiplexed sensing, and signal reconstruction methods, which can reduce communication and computation burdens without relying solely on stronger interface standardization.

In addition to structural modularity, the generalization of dexterous hand systems depends on the coordinated integration of sensing, actuation, and control subsystems. However, current systems often suffer from nonstandardized interfaces, conflicts between sensor wiring and actuator layout, and inconsistencies in power supply and communication protocols. These issues hinder data transmission between modules and reduce deployment efficiency. To address such limitations, some systems have adopted unified communication strategies.

To further mitigate integration challenges, some studies explore coordinated hardware–software integration at the communication level. However, codesign in a broader sense should not be reduced to interface unification alone, because high-performance dexterous hands often require the joint optimization of embodiment, sensing, and control at the system level. Communication architecture represents a critical but still underexplored aspect of dexterous hand system integration. While distributed systems such as CAN are sometimes adopted in robotic platforms, their suitability for dexterous hands depends on factors such as data bandwidth, synchronization requirements, and latency constraints, particularly in systems with dense tactile sensing and high-frequency control loops. At present, no widely accepted communication standard exists for dexterous hand systems, and protocol selection remains closely tied to specific system architectures and application requirements.

More fundamentally, the current lack of standardization in dexterous hands reflects the absence of converged solutions across key subsystems, including sensing architectures, actuation mechanisms, and control strategies. Different design choices lead to fundamentally different system requirements, making it difficult to define universal interface standards. Therefore, standardization should be viewed not as an immediate solution but as a longer-term outcome that may emerge as the field matures and dominant system architectures become established.

Recent advances have improved the modular structure and interface integration of dexterous hand systems. However, key challenges remain that hinder their large-scale deployment. Task variability imposes diverse demands on module functionality and structural adaptability, which promotes the development of reconfigurable and open architectures. At the same time, the separation of hardware and software components has led to a shift toward distributed system frameworks. This evolution requires unified communication protocols, consistent identification schemes, and clearly defined control semantics between modules. Future systems may benefit from task-driven modular design and improved interface definitions. However, achieving meaningful standardization will require advances in sensing, actuation, and control technologies, as well as a better understanding of system-level trade-offs. Progress in dexterous hand deployment is therefore likely to depend not only on interface design but also on the codevelopment of robust and scalable subsystem technologies.

### Strategy-coupled control driven by multimodal perception

As tasks become more complex and environments more dynamic, dexterous hand systems are moving away from single-sensor explicit control. In response, the control architecture is evolving toward strategy generation mechanisms that integrate information from multiple sensing modalities. In traditional designs, the perception–control pipeline is typically built on manual modeling and rule-based mappings. These methods focus on capturing physical variables accurately and maintaining closed-loop feedback. However, they are not well suited to real-world conditions, where sensor data can be uncertain, diverse, and high-dimensional. These limitations reduce the generalization ability and robustness of the system, prompting a shift toward more integrated and adaptive control strategies.

Recent research shows that combining sensing and control across multiple modalities helps systems interpret tasks and adapt to change. Transformer models have been used to integrate tactile, visual, and semantic information. This enables decision-making and adaptive control under complex tasks. Some approaches link visual input, task objectives, and motor actions, allowing the system to recognize task intentions and coordinate motion planning. Other studies explore frameworks that combine large language models with reinforcement learning. These systems can generate complex behaviors without relying on precise models of the environment, marking a departure from traditional rule-based control [104].

Despite its promising potential, this direction still faces several challenges at the system level. First, the heterogeneity of multimodal inputs in temporal and spatial resolution makes data alignment and fusion difficult. Second, delays in the perception-to-decision loop can compromise the stability of strategy execution. Third, the computational demands of large-scale models limit their ability to run in real time, especially on edge platforms. Balancing accuracy, response speed, and resource consumption remains a key issue for the practical implementation of integrated perception–control frameworks [105,106].

### Design of trustworthy human–hand interaction mechanisms

Dexterous hands are gradually expanding from research settings into real-world application sectors such as intelligent manufacturing, medical rehabilitation, and human–robot collaboration. These sectors do not merely provide future use cases; they actively shape the technical development of dexterous hand systems by imposing different performance priorities. In intelligent manufacturing, dexterous hands must support repeatable and efficient operation under workflow constraints, including stable grasp execution, robustness to part variation, compatibility with cycle-time requirements, and maintainability in production settings. In medical rehabilitation, the dominant requirements shift toward compliance, comfort, long-term safety, patient-specific adaptation, and trust during sustained physical interaction. In human–robot collaboration, dexterous hands must combine physical safety with interpretable behavior, intention-aware interaction, and reliable task adaptation in shared and changing environments. Soft robotic integration is particularly relevant in prosthetic reconstruction and rehabilitation, where compliant contact reduces interaction risk, lightweight structures improve wearing comfort, and sensory feedback can improve user embodiment and confidence during manipulation [13–15].

These sector-specific requirements also reveal distinct remaining challenges. In intelligent manufacturing, the central difficulty is not only achieving force and positional accuracy but balancing dexterity with repeatability, cycle-time efficiency, fault tolerance, and deployment robustness under practical industrial constraints. In medical rehabilitation, even though compliant mechanisms and intention-aware interaction strategies have improved safety and usability, major challenges remain in long-term personalization, comfort–function trade-offs, clinical robustness, and trust formation over extended use. In human–robot collaboration, existing work has improved safety boundary modeling, decision visualization, and interactive feedback correction, but important issues remain in interpretable decision-making, multimodal intent understanding, adaptive yet predictable behavior, and trust calibration during long-term interaction. These challenges indicate that application-driven dexterous hand development is still constrained not only by hardware capability but also by how reliably the system can align technical performance with user expectations and task context [107,108].

### Future challenges and opportunities for deployable dexterous hand systems

The future development of deployable dexterous hand systems can be understood most clearly by returning to the 3 key challenges identified in Introduction: system complexity, limited task generalization, and the integration of sensing and control. These challenges also correspond to the technical tensions highlighted in Fig. 1, where embodiment, perception, control, and deployment-oriented requirements interact across multiple system layers. Accordingly, this final section does not merely outline broad future prospects but instead discusses the remaining bottlenecks and corresponding opportunities for dexterous hands along these 3 challenge axes.

#### System complexity: From component proliferation to coordinated system architecture

A first major challenge is system complexity, which increases rapidly as dexterous hands incorporate high-degree-of-freedom structures, multimodal sensing, distributed actuation, and embedded computation. In practice, this complexity leads not only to hardware integration difficulty but also to packaging constraints, calibration burden, maintenance cost, and reduced deployment reliability. The corresponding opportunity lies in moving from isolated component optimization toward coordinated system architecture. Task-aware modularity, compact hardware–software codesign, unified communication and calibration frameworks, and diagnostic-friendly packaging are all promising directions for managing complexity without sacrificing dexterity.

#### Limited task generalization: From task-specific policies to scalable embodied learning

A second challenge is limited task generalization. Although many dexterous hand systems perform well in constrained demonstrations, their policies often degrade when contact conditions, object geometry, task sequence, or environment distribution changes. This limitation reflects not only the difficulty of learning robust manipulation skills but also the sensitivity of dexterous behavior to embodiment and sensing conditions. Future opportunities therefore lie in scalable embodied learning, including multimodal representations that couple tactile, visual, and semantic information, large-scale human-to-robot transfer, world-model-based prediction, and task-adaptive policy learning. These routes may help dexterous hands move from narrow task execution toward more transferable and reusable manipulation competence [109].

#### Integration of sensing and control: From perception pipelines to perception–action coupling

A third challenge is the integration of sensing and control, which remains a core bottleneck for dexterous hands operating in dynamic and uncertain environments. In current systems, perception and action are still often linked through loosely coupled or sequential pipelines, making it difficult to maintain stable feedback under multimodal latency, sensing heterogeneity, and complex contact transitions. The key opportunity here is to develop more tightly coupled perception–action architectures, in which proprioceptive, tactile, visual, and semantic streams are integrated into shared representations that directly support adaptive policy generation and safe control execution. In this sense, future progress depends not only on better sensors or stronger controllers in isolation but also on how effectively sensing and control are codesigned as a unified closed-loop system [110–112].

Viewed together with Fig. 1, these future directions show that the next stage of dexterous hand development will depend on how effectively structural embodiment, multimodal perception, control, and deployment-oriented integration can be advanced in a coordinated manner. Progress in modular design, multimodal control, and trustworthy human–hand interaction is improving generalization, adaptability, and collaborative capability, while directly addressing long-standing bottlenecks in deployment efficiency, robust control, and user integration. This trajectory signals a shift from hands as task-specific tools to hands as integrated interactive agents. Continued advances will require stronger semantic understanding, tighter integration of information across sensing and control, and user-centered collaboration that remains reliable under uncertainty. A unified hardware–software framework with standardized interfaces will be essential for scalability and maintainability. As technical maturity aligns with practical demands, broader adoption is expected in manufacturing, rehabilitation, and service robotics, supporting more reliable and effective human–robot collaboration.

This review summarizes recent progress in dexterous hands for complex manipulation and contributes a system-level framework for understanding their development and deployment. The main contribution of the review is 3-fold. First, it organizes dexterous hand technologies around 4 tightly coupled dimensions: structural embodiment, multimodal perception, control, and deployment-oriented integration. Second, it highlights the cross-layer dependencies among morphology, sensing, decision-making, and deployability, showing why advances in individual components do not necessarily translate into robust system-level performance. Third, it identifies system complexity, limited task generalization, and sensing–control integration as major challenges that shape future dexterous hand development. In this way, the review provides not only a summary of representative technologies but also an analytical framework for evaluating and guiding future dexterous hand systems.
